# Supplementary material for: Novel candidates of pathogenic variants of the BRCA1 and BRCA2 genes from a dataset of 3,552 Japanese whole genomes (3.5KJPNv2)
Source: PLoS One. 2021 Jan 11;16(1):e0236907. doi: 10.1371/journal.pone.0236907 (PMC7799847; doi:10.1371/journal.pone.0236907)
Supplement: Attachment — Submitted filename: Response_to_Reviewers.docx [file pone.0236907.s007.docx]

PONE-D-20-21851R1
Novel candidates of pathogenic variants of the BRCA1 and BRCA2 genes from a dataset of 3,552 Japanese whole genomes (3.5KJPNv2)

6. Review Comments to the Author

Please use the space provided to explain your answers to the questions above. You may also include additional comments for the author, including concerns about dual publication, research ethics, or publication ethics. (Please upload your review as an attachment if it exceeds 20,000 characters)

Reviewer #1: The authors’ efforts are appreciated with regard to their revisions on the manuscript. This has resulted in text that is significantly improved, but continues to have some ongoing issues towards publication.

MAJOR: (1) With regard to InterVar use, to reiterate/clarify the point from the initial review, the authors did not explain their handling of noncoding variants (which is, as they clarified, is the point of WGS data over exonic data). Intervar describes handling of the mutation types in question as follows:

*Splicing -> Intervar does address some splicing variants, but they describe in the Li paper that their data cleaning procedure includes removal of variants with conflicting interpretation as part of data cleaning prior to checking the dbscsnv11 database. (This is confusing as the authors then show variants that have conflicting interpretations in Clinvar? I wasn't clear how these would have been identified if Intervar was used as described in the Li paper?)

Thank you very much for detailed comment. To answer to the point, we checked the Intervar annotation data again and we did not find any variants that may affect splicing but interpreted as “Benign” or “likely benign” in the *BRCA1/2* gene variants in 3.5KJPN. There is one variant, the BRCA2 I2675V, that is known as a splicing error-causing variant in 3.5KJPN and the variant is annotated as P or LP by both Clinvar and Intervar. To clarify the issue, we added following sentences in the “Results and discussions” section (p22).

“So far there is no other splicing error-causing variants of the *BRCA1/2* genes in the 3.5KJPN. As shown in Table 1, there are two splicing-affected variants (BRCA2:g.chr13: 32890558AGdel and BRCA2:g.chr13: 32937315G>A) in the GnomAD-EAS, indicating that we did not miss a large numbers of splicing error causing variants in the *BRCA1/2* genes in the 3.5KJPN.”

*Intronic / noncoding variants -> This point was not addressed by the authors although the WGS would allow them to do so (and that is noted as one of their reasons for novelty). ANNOVAR identifies intergenic and noncoding variants, but InterVar’s web application is specifically designed for annotation use in exons and their paper notes that:
“InterVar is designed to interpret genetic variants that are likely to cause Mendelian diseases or are highly penetrant for Mendelian diseases (OR > 5) and cannot handle alleles that increase susceptibility to common and complex traits. Therefore, we caution that the current interpretation is appropriate only for Mendelian diseases or Mendelian forms of complex diseases.”

If the authors intend to only address splicing using WGS data over the existing exonic data, then region of application is what needs to be clarified consistently through the paper and a point should be made in the discussion regarding limitations of InterVar in this context.

If they intend to state that they analyzed intronic / noncoding variants related to BRCA1/BRCA2, then it is not clear that InterVar as described with use of default settings is a good tool for intronic / noncoding variants. I would refer the authors to the literature for references such as this: https://www.ncbi.nlm.nih.gov/pmc/articles/PMC6266896/ It is noted that the variant annotation, albeit different, cited by the authors in their prior analysis on 2KJPN demonstrates ability to pursue this work – this point is just not clear.

Thank you very much for the comment and we apologize our unclear explanations. We have used command-line based InterVar software with its default option. It can annotate the noncoding variants of a gene and intergenic regions, too. As we mentioned in the methods section of our manuscript, the command-line Intervar is depends on the ANNOVAR function so that it can cover non-coding variants. In the *BRCA1/2* genes in the 3.5KJPN, there are 2891 non-coding (intronic, 5’-UTR, and 3’-UTR) variants and all of them are annotated by the software. There is no P or LP variants in either Clinvar or Intervar. To clarify the issue, we added the following sentences in the “Results and discussions” section (p17).

Hence, InterVar may underestimate the clinical impact of potentially pathogenic variants about which previous information is not available. The tendency might be worse in the noncoding regions in the coding genes like the BRCA1/2 genes because of the lack of functional studies for such regions. Nowadays, the whole genome sequencing data is accumulating and comparisons between the phenotypes and variants in the noncoding regions found by the WGS will provide critical data for the interpretation of the noncoding variants.”

(2) There continue to be problems with conflation of moderate penetrance and likely pathogenic variants that are concerning. The term “moderately pathogenic variants” remains unclear. Please revise “moderately pathogenic” to “likely pathogenic” (related to variants) or “moderate penetrance” (related to genes) depending on what was intended as these are the standard accepted terms in the field.

For instance, these descriptions are completely inaccurate regarding management of patients with BRCA1/BRCA2 mutations because of this conflation problem.
“The carriers of moderately pathogenic HBOC variants will not have
undergone the drastic prophylactic modalities but frequent examination will be recommendable for earlier detection of the cancers.”
“The presence of such variants may not be critical to prompt radical interventions such as
prophylactic surgery, but the carriers may be encouraged to continue undergoing close health checks to detect HBOC cancers as early as possible.”

Thank you very much for the comments. We understand the reviewer’s criticism as a word “pathogenic” should not be used for a variant with low susceptibility that may not be needed any medical drastic actions. We would like to use “likely pathogenic” for variants that can cause diseases with enough high provability for medical action and “moderate” variants that shows low but significantly susceptible for disease onset. So, we amended the issue throughout the manuscript not to use “pathogenic” for explanation of such weakly disease-causing variants. In terms of the two exemplified sentences, we amended as follows:

“The carriers of moderately deleterious HBOC variants would not undergo drastic prophylactic modalities, but frequent examination would be recommendable for earlier detection of the cancers.”

“The carriers in moderately penetrant HBOC families may not be critical to prompt radical interventions such as prophylactic surgery, but the carriers may be encouraged to continue undergoing close health checks to detect HBOC cancers as early as possible.”

Also:
“Many of the cancer-predisposing genes are known to be associated with juvenile cancer
syndromes such as Li Fraumeni syndrome. The variants responsible for juvenile cancer syndromes
are usually very pathogenic and show strong effects on gene functions.”

Li Fraumeni syndrome is a specific diagnosis associated with a specific gene, not multiple genes. These sentences in particular should just be removed completely.

Thank you very much for critical comment and we accept the request and deleted the sentences.

(3) If part of the purpose of the paper is to evaluate the performance of InterVar in annotation, Clinvar and InterVar variants should be treated as separate categories. There are multiple parts of the results in which variants from these groups are just put together.

Thank you very much for the careful comments and we apologize the confusing way of our explanation. We did not include the evaluation of performance of InterVar in annotation. We just use it to supplement the lack of annotation by Clinvar and obtain the info of the other parameters such as CADD, DAN, and Eigen. Most of the conflicts between two databases are caused by the lack of information in Clinvar. So, we use them basically the same manner.

MINOR:
Introduction:
-On page 4, in the introduction, the term “approaches” is unclear since what the authors are referring to is that there are several efforts (whereas approaches implies different strategies of ascertaining pathogenicity of identified mutations).

Thank you very much for careful comments and we amended the sentences to clarify our intention as follows:

“Several levels of studies (single organization, single nation, and international level) have been done previously. As a single organization study, Sugano et al. reported the *BRCA1* and *BRCA2* germline variants in 135 HBOC patients and identified 28 pathogenic ones [9]. As the nationwide study, Arai et al. examined 830 Japanese HBOC pedigrees collected by the Japanese HBOC consortium and identified 49 different pathogenic variants among them [10]. Similarly, a nationwide multicenter study revealed that germline *BRCA* 1/2 mutations were present in 14.7% of 634 Japanese women with ovarian cancer [5]. Lee et al. also examined the variants in the *BRCA1* and *BRCA2* genes in breast and ovarian cancer patients’ germline genomic DNA and calculated posterior probabilities for the disease-causing mutations; they identified five previously unreported variants as candidate pathogenic ones [11]. Finally, as an international study, BRCA Exchange…”

-Would modify result/discussion subtitles from “Pathogenic variants in the two…” -> “Estimation of pathogenic variants…” and “Estimate of computational scoring tools for pathogenicity of the 3.5KJPNv2 BRCA variants” -> “Estimate of computational scoring tools’ performance in predicting pathogenicity of novel 3.5KJPNv2 BRCA variants”

Thank you very much for critical comment and we accept the request and amended as suggested.

-Please revise references to genomAD for spelling/capitalization accuracy in the Introduction.

Thank you so much for the careful comment and we amended the issue throughout the manuscript as “gnomAD”.

-Methods:
-The context for the statement in the methods regarding candidate variants in the Korean population is not clear.

Thank you very much for the comment. We tried to fix the issue as following:

“The positions of the candidate pathological variants found in the Korean population [11] were described as the cDNA positions. Ti apply the data to the InterVar software, …“

-Continuing to have a lot of difficulty understanding how the authors were able to link information from the familial TMM database / questionnaire with the sequencing information if these are located in two separate datasets w/ two separate accesses.

Thank you for the comment and we apologize the unclear explanation. So, we added the following explanation in the Methods section.

“The TMM database is a relational database and it consists of several separate datasets. The key is the participants’ IDs to link the information stored in the different tables”

-Results/Discussion
The authors’ note about cancer-bearing offspring mentions p=0.041 is not statistically significant but does not specify what threshold would be significant – presumably with a Chi-squared test this would be 0.05, so this is statistically significant?). However, if there was a correction done for multiple testing (which would be appropriate if testing was done across numerous variants), then this is not clear from the paper as written.

Thank you very much for the constructive comments. We felt that the 0.041 was not enough low to be “significant”. As the reviewer suggested, it should be interpreted as “marginally significant” if the p < 0.05 is the threshold. So we amended the main text as follows. To fulfil the requirement of the PLoS One policy for publication, we added the data for offspring cancer burden in the TMM database as Supplementary Table 5 and fixed Figure 2 accordingly. So, the figure legend of Figure 2 also amended.

“A prominent difference between those definitely carrying potentially pathogenic BRCA variants and the rest of the cohort was in the rate of cancer-bearing sisters: the InterVar P or LP carriers were shown to have a much higher rate of cancer-bearing sisters than the rest of the cohort (Fig. 2 and Supplementary Table 5; p = 3.08 × 10−5, chi-squared test with Yates’ correction). In addition, the rate of cancer-bearing offspring was higher in the InterVar P or LP carriers than in the others with marginally significant (p = 0.041).”

Figure 2 legend:

“…Asterisks indicate statistically significant differences (single: p < 0.05, double: p < 10-4) upon comparison with the total analyzed TMM CommCohort cases (Fig. 2).”

Reviewer #2: (No Response)

7. PLOS authors have the option to publish the peer review history of their article ([what does this mean?](https://journals.plos.org/plosone/s/editorial-and-peer-review-process#loc-peer-review-history)). If published, this will include your full peer review and any attached files.


If you choose “no”, your identity will remain anonymous but your review may still be made public.


**Do you want your identity to be public for this peer review?** For information about this choice, including consent withdrawal, please see our [Privacy Policy](https://www.plos.org/privacy-policy).

Reviewer #1: No

Reviewer #2: No

[NOTE: If reviewer comments were submitted as an attachment file, they will be attached to this email and accessible via the submission site. Please log into your account, locate the manuscript record, and check for the action link "View Attachments". If this link does not appear, there are no attachment files.]

While revising your submission, please upload your figure files to the Preflight Analysis and Conversion Engine (PACE) digital diagnostic tool, <https://pacev2.apexcovantage.com/>. PACE helps ensure that figures meet PLOS requirements. To use PACE, you must first register as a user. Registration is free. Then, login and navigate to the UPLOAD tab, where you will find detailed instructions on how to use the tool. If you encounter any issues or have any questions when using PACE, please email PLOS at [figures@plos.org](mailto:figures@plos.org). Please note that Supporting Information files do not need this step.

*In compliance with data protection regulations, you may request that we remove your personal registration details at any time.* [*(Remove my information/details)*](https://www.editorialmanager.com/pone/login.asp?a=r)*. Please contact the publication office if you have any questions.*
